# Do patients with gastroesophageal reflux disease exhibit compromised bone quality prior to proton pump inhibitor therapy?

**DOI:** 10.1016/j.bonr.2021.101095

**Published:** 2021-05-20

**Authors:** Kristin M. Aasarød, Mats P. Mosti, Malin T. Finstad, Astrid K. Stunes, Reidar Fossmark, Unni Syversen

**Affiliations:** a Department of Clinical and Molecular Medicine, Norwegian University of Science and Technology, Trondheim, Norway; b Department of Gastroenterology and Hepatology, St. Olavs Hospital, Trondheim, Norway; c Medical Clinic, St. Olavs University Hospital, Trondheim, Norway; d Department of Endocrinology, St. Olavs Hospital, Trondheim, Norway

**Keywords:** Proton pump inhibitors, Bone mineral density, Trabecular bone score, Bone metabolism, Gastrin

## Abstract

Patients with gastroesophageal reflux disease (GERD) are routinely treated with proton pump inhibitors (PPIs), despite many reports of increased fracture risk associated with PPI use. Notably, the skeletal properties in patients with GERD prior to PPI therapy have not been addressed. We hypothesized that PPI-naïve GERD patients have bone impairment, and that short-term treatment with PPI has minimal skeletal effects. To test this, 17 (12 men/5 women) GERD patients age 32–73 years, not previously exposed to PPI, and 17 age- and sex-matched controls were enrolled from September 2010 to December 2012. Bone mineral density (BMD) at lumbar spine, femoral neck, total hip, and trabecular bone score (TBS) at the lumbar spine, a marker of bone microarchitecture, were measured by dual X-ray absorptiometry. Markers of bone turnover and calcium homeostasis, and gastric hormones were analyzed. The same parameters were measured after three months of treatment with the PPI pantoprazole. The GERD patients displayed a significantly lower TBS at baseline than controls (1.31 ± 0.11 vs. 1.43 ± 0.07, p = 0.0006). Total hip and femoral neck BMD were lower in patients compared to controls, however, not significantly (p = 0.09 and 0.12, respectively). CTX was non-significantly higher in GERD patients at baseline (p = 0.11). After three months, changes in BMD, TBS and CTX did not differ between the groups. In conclusion, this is the first report demonstrating compromised bone quality and inferior BMD in PPI-naïve GERD patients. Treatment with pantoprazole did not influence bone parameters, indicating that short-term use with this PPI is safe for the skeleton.

## Highlights

• Previous studies have reported an increased fracture risk in PPI users.
• Patients with GERD had compromised bone quality before starting PPI.
• Three months treatment with pantoprazole did not influence bone parameters.

## 1 Introduction

Gastroesophageal reflux disease (GERD) is prevalent worldwide. A systematic review showed a prevalence of 18.1–27.8% in North America and 8.8–25.9% in Europe (Yamasaki et al., 2018). Over the last decades, there has been a significant increase in the proportion of younger patients with GERD, especially those within the age range of 30–39 years. GERD is often treated with proton pump inhibitors (PPIs), which are among the most prescribed drugs worldwide. PPIs have gained attention as several observational studies have suggested that PPI use is an independent risk factor for fractures (Targownik et al., 2008; Vestergaard et al., 2006; Yang et al., 2006; Gray et al., 2010). Whether patients with GERD have skeletal impairment before exposure to PPIs has, however, not been properly addressed. Accordingly, there are no reports on fracture risk, bone mineral density (BMD) or bone quality in PPI-naïve GERD patients.

Data on the effect of PPIs on fracture risk have been diverging. A large meta-analysis by Poly et al. from 2019 including 24 observational studies and 2,103,800 participants reported a modestly increased risk of hip fractures in those treated with PPIs (RR = 1.22) (Poly et al., 2019). The fracture risk seemed to depend on type of PPI, with an increase by 27% in users of rabeprazole and 13% in users of omeprazole and pantoprazole (Poly et al., 2019). On the other hand, use of esomeprazole and lansoprazole was not associated with increased hip fracture risk. In a study applying data from the Nord-Trøndelag Health Study (HUNT3), including 15,017 women and 13,241 men aged 50–85 years, no increase in risk of fractures was observed (Hoff et al., 2020).

Fracture risk is determined by several factors, including BMD, bone quality, and bone turnover. Studies in rodents and humans have demonstrated reduced BMD after PPI use, whereas others have failed to show a reduction in BMD. Attenuated BMD was observed in rats given omeprazole for 3 months (Cui et al., 2001) and in H+/K+-ATPase deficient mice (Aasarod et al., 2016a; Aasarod et al., 2016b; Fossmark et al., 2012). Notably, two studies observed no significant bone loss after ten years of PPI treatment (Targownik et al., 2012; Solomon et al., 2015), whereas others showed a significant, but small decline in BMD after 6 months to 3 years (Gray et al., 2010; Ozdil et al., 2013; Bahtiri et al., 2016). A systematic review and meta-analysis from 2020 reported a reduction in BMD among PPI users regarding the mean difference in BMD (Aleraij et al., 2020). However, no significant association between PPI users and nonusers with respect to the mean annualized percent change in BMD was observed (Aleraij et al., 2020).

BMD measured by dual X-ray absorptiometry (DXA) is the gold standard indiagnosis of osteoporosis and prediction of fracture risk. It should be recalled that BMD gives an estimation of bone quantity, but provides no information on bone quality, another determinant of bone fragility. Bone quality encompasses structural and material properties of bone (Chavassieux et al., 2007; Seeman and Delmas, 2006).

Trabecular bone score (TBS) is a surrogate measure of microarchitecture based on grey-level texture of spine DXA images, which has been shown to improve the prediction of fracture risk in combination with BMD (Hans et al., 2011). So far only one study has assessed TBS in PPI users. Shin et al. reported lower TBS in subjects with PPI exposure than in controls (Shin et al., 2019).

Numerous studies have explored potential harmful skeletal effects of PPIs, whereas data on bone health are lacking among PPI-naïve GERD patients. Hence, we aimed to investigate BMD, TBS and bone turnover in patients with GERD not exposed to PPIs previously compared to healthy, age-matched controls. Moreover, we wanted to assess whether these parameters were influenced by treatment with the PPI pantoprazole for three months.

## 2 Methods

### 2.1 Study design and participants

Inclusion criteria were: PPI-naïve patients of both sexes, aged 25–80 years, with endoscopically confirmed GERD, and planned treatment with pantoprazole 40 mg o.d. for at least three months. Esophagitis was graded according to the Los Angeles (LA) classification (Armstrong et al., 1996). The patients were recruited between September 2010 to December 2012 from the Endoscopy unit at the Department of Gastroenterology and Hepatology at St. Olavs Hospital, Trondheim, Norway. Age- and sex-matched controls were recruited from registered blood donors and by advertising on the intranet webpage of the Norwegian University of Science and Technology (NTNU). Exclusion criteria were previous fractures at adult age or known osteoporosis. Furthermore, we excluded patients using medications known to affect bone metabolism (i.e. glucocorticoids, antiepileptics). Neither patients nor controls got supplements with B12. The patients were given oral and written information and gave written consent to participate. The study was approved by the Regional Ethics Committee.

### 2.2 Measurement of BMD and TBS by dual X-ray absorptiometry (DXA)

BMD at the lumbar spine (L1-L4), total hip and femoral neck was measured by DXA Hologic Discovery A (S/N 83817). BMD (g/cm2) and *Z*-scores were used for group comparisons. Trabecular bone score (TBS) of the lumbar spine (L1-L4) was calculated using the TBS iNsight® software version 2.1.0.0 (Medimaps, Pessac, France). The same operator performed the DXA measurements and analyzed all scans at the end of the study. The coefficients of variations for BMD were 1.1% at the lumbar spine, 1.3% at the total hip, and 1.5% at the femoral neck.

### 2.3 Serum, plasma and urine analyses

Fasting blood samples were collected at baseline and after three months, and serum and plasma samples were stored at −80 °C until analyses. Blood leukocytes and erythrocyte sedimentation rate (ESR), C-reactive protein (CRP) and creatinine were measured at baseline only. The other parameters were analyzed both at baseline and after three months. Procollagen type 1 amino-terminal propeptide (P1NP) and C-terminal telopeptide **(**CTX) were analyzed by electrochemoluminescence immunoassays at the Hormone Laboratory, Oslo University hospital (Oslo, Norway). Additionally, the following parameters were measured in serum or plasma: alkaline phosphatase (ALP), albumin, vitamin D 25-OH, and parathyroid hormone (PTH). Magnesium, calcium and creatinine were measured in morning urine samples. All analyses were performed by routine methods at the Department of Clinical Chemistry, St. Olavs Hospital (Trondheim, Norway). Gastrin was analyzed by radioimmunoassay (RIA) as described previously (Kleveland et al., 1985), and chromogranin A (CgA) by a RIA (Euria-Chromogranin A, Eurodiagnostika, Malmö, Sweden).

### 2.4 Statistics

Data are presented as mean ± one standard deviation (SD) or median (interquartile range (Q1-Q3)), depending on data distribution, which was assessed with the Kolmogorov-Smirnov test for normality. Baseline data in the two groups were compared using either a two-sided student's *t*-test, or the Mann-Whitney *U* test, depending on data distribution. Changes from baseline (delta values) between the two groups were analyzed with a univariate linear regression model with baseline values as covariate (ANCOVA) for normally distributed data. For nonparametric data sets, delta values were evaluated by the Mann-Whitney *U* test. A p-value <0.05 was considered significant. A paired samples *t*-test was performed to examine changes from baseline within one or both of the groups. All analyses were performed using IBM SPSS statistics version 22.0.

### 2.5 Results

Twelve men and five women, mean age 54 ± 11 years (range 32 to 73 years), with endoscopically verified GERD, and 17 age- and sex-matched, healthy controls 51 ± 10 years were included. Most patients with GERD had either LA grade A (n = 8) or B (n = 8) esophagitis. BMI did not differ between the groups. Leukocyte counts and ESR were significantly higher in the patient group (Table 1).

**Table 1:** Baseline characteristics of patients with GERD and controls.

Variable	Patients n = 17	Controls n = 17	Reference range
Male/female (n)	12/5	12/5	
Age (years)	54 ± 11	51 ± 10	
BMI (kg/m^2^)	25.7 ± 2.6	25.8 ± 7.5	
S-CRP (mg/L)	<5	<5	<5
ESR (mm)	7 (4–12)^a^	2 (2–8)	1–19
Leukocytes (10^9^/L)	5.9 ± 2.0^b^	4.6 ± 1.1	3.7–10.0
S-Creatinine (μmol/L)	69 ± 13.9	77 ± 12.6	60–100
*H. pylori* positive (n)	1	2	
Esophagitis LA grade (n)			
A	8	–	
B	8	–	
C	1	–	
D	0	–	

Data are presented either as mean ± SD or median (IQR), depending on data distribution. ^a^p = 0.049, ^b^p = 0.014. GERD: gastroesophageal reflux disease; BMI: body mass index; S: serum; CRP: C-reactive protein; ESR: erythrocyte sedimentation rate; LA: Los Angeles.

### 2.6 BMD and TBS

At baseline lumbar spine BMD did not differ between GERD patients and controls (1.062 ± 0.23 g/cm^2^ vs. 1.120 ± 0.18 g/cm^2^, p = 0.84). There was a trend towards lower total hip BMD in GERD patients compared to controls (0.946 ± 0.16 g/cm^2^ vs. 1.038 ± 0.15 g/cm^2^, respectively, p = 0.09) and a similar trend was observed for femoral neck BMD (0.794 ± 0.14 g/cm^2^ vs. 0.871 ± 0.14 g/cm^2^, p = 0.12). Similar trends were observed for *Z*-scores, with lower Z-score for total hip in GERD patients than in controls (0.076 ± 1.02 vs. 0.682 ± 1.01, p = 0.09). The GERD patients displayed a significantly lower TBS at baseline than controls (1.31 ± 0.11 vs. 1.43 ± 0.07, p = 0.0006). Data are presented in Table 2.

**Table 2:** Bone mineral density, BMD *Z*-score and trabecular bone score at baseline and after three months of PPI treatment.

	Baseline		Change	
	Patients n = 17	Controls n = 17	Patients n = 17	Controls n = 17
BMD lumbar spine (g/cm^2^)	1.062 ± 0.23	1.120 ± 0.18	−0.011 ± 0.03	−0.002 ± 0.03
BMD femoral neck (g/cm^2^)	0.794 ± 0.14	0.871 ± 0.14	0.003 ± 0.02	0.002 ± 0.02
BMD total hip (g/cm^2^)	0.946 ± 0.16	1.038 ± 0.15	0.015 ± 0.19	0.006 ± 0.023
BMD Z-score lumbar spine	0.441 ± 2.00	0.971 ± 1.80	0.00 ± 0.46	−0.24 ± 0.26
BMD Z-score femoral neck	0.029 ± 1.01	0.553 ± 1.13	−0.15 ± 0.68	0.01 ± 0.19
BMD Z-score total hip	0.076 ± 1.03	0.682 ± 1.01	−0.54 ± 1.27	0.04 ± 0.14
TBS (L1-L4)	1.31 ± 0.11^a^	1.43 ± 0.07	0.00 ± 0.05	0.00 ± 0.04

Data are presented as mean ± SD. BMD: bone mineral density; PPI: proton pump inhibitor; TBS: trabecular bone score. ^a^p = 0.001.

After three months of PPI use, a non-significant decrease in BMD at the lumbar spine was observed. The alteration in BMD was not significantly different from the control group (p = 0.39). Femoral neck BMD in both groups remained unchanged. Total hip BMD increased slightly in the patient group, but the gain did not differ significantly from the control group (Table 2). TBS did not change between baseline and three months within or between the groups.

### 2.7 Serum and plasma and urine analyses

At baseline, CTX was higher in the GERD patients compared to controls, although not significantly (0.56 ± 0.25 ng/mL vs. 0.43 ± 0.24 ng/mL, p = 0.13); ALP and P1NP levels did not differ between the groups (Table 3). Vitamin B12 levels were significantly higher in the control group (Table 1), (271.6 ± 78.6 pmol/L vs. 339.9 ± 93.5 pmol/L). The GERD patients had higher PTH levels compared to controls (5.7 (4.3–7.0) pmol/mL vs. 4.1 (3.5–5.0) pmol/mL, p = 0.025) and a trend towards higher vitamin D 25-OH (74.8 ± 26.9 nmol/L vs. 58.4 ± 22.5 pmol/L, p = 0.063).

**Table 3:** Bone markers at baseline and after three months of PPI treatment.

	Baseline		Change		
	Patients n = 17	Controls n = 17	Patients n = 17	Controls n = 17	Reference range
S-CTX (ng/mL)	0.56 (0.44–0.69)	0.43 (0.36–0.60)	−0.01 (−0.07–0.06)	−0.02 (−0.08–0.06)	*
S-P1NP (μg/L)	45 (36.5–50.5)	44 (39.5–57.0)	6 (1–1.5-10.5)	0 (−3–4)	♀11–94 μg/L ♂20–91 μg/L
S-ALP (U/L)	63.2 ± 15.6	59.8 ± 19.0	0.9 ± 9.3	3.6 ± 4.9	35–105 U/L

Data are presented either as mean ± SD or median (IQR) depending on distribution. *reference ranges depend on gender and age: ♀ 25–49 years: ≤0.57 ng/ml, ≥50 years ≤1.01 ng/ml. ♂ 30–50 years ≤0,58 ng/ml, 51–70 years ≤0.70 ng/ml, ≥70 years ≤0,85 ng/ml. PPI; proton pump inhibitor; S: serum; P1NP: Procollagen type 1 amino-terminal propeptide; CTX: C-terminal telopeptide; ALP: alkaline phosphatase.

All serum analyses were, however, within the reference range at baseline. Calcium and magnesium levels in urine were similar in the two groups.

**Fig. 1:**
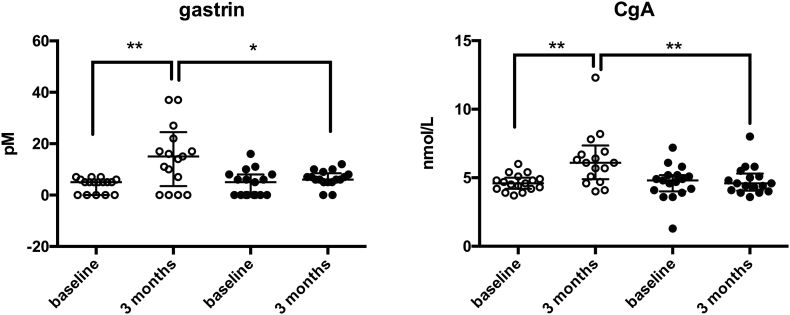
Serum gastrin and chromogranin A (CgA) in patients and controls at baseline and three months. One outlier in the patient group at three months was left out to make the figure more readable. (outlier values: gastrin 92, CgA 35). ○ patients ● controls * p < 0.05 ** p = 0.01 CgA: Chromogranin A.

After three months, gastrin and CgA increased significantly in the patient group compared to controls. A rise in vitamin B12 was observed in the control group, as well as an increase in vitamin D 25-OH level. PTH levels did not change significantly in any of the groups. Delta values for other serum parameters did not differ significantly between the groups. Changes in urine concentrations of calcium and magnesium adjusted for urine creatinine were not significantly different between the groups. Data are presented in Table 4 and Fig. 1.

**Table 4:** Parameters of calcium and magnesium homeostasis in blood and urine at baseline, and after three months of PPI treatment.

	Baseline		Change		
	Patients n = 17	Controls n = 17	Patients n = 17	Controls n = 17	References range
S-Ion Ca (mmol/L)	1.22 ± 0.03	1.23 ± 0.02	−0.01 ± 0.06	0.02 ± 0.04	1.18–1.32
P-Phosphate (mmol/L)	1.00 (0.25)	1.00 (0.25)	0.04 (0.25)	0.05 (0.21)	0.71–1.23
P-Magnesium (mmol/L)	0.82 ± 0.05	0.82 ± 0.04	−0.006 ± 0.040	−0.007 ± 0.033	0.71–0.94
S-PTH (pmol/mL)	5.7 (4.3–7.0)^a^	4.10 (3.5–5.0)	−0.6 (−1.9–0.8)	−0.7 (−1.2–0.2)	1.3–6.9
S-vitamin B12 (pmol/L)	271.6 ± 78.6^b^	339.9 ± 93.5	−3.4 ± 74.4	48.6 ± 42.7^c^	141–489
S-vitamin D 25-OH (nmol/L)	74.8 ± 26.9	58.4 ± 22.5	−6.4 ± 21.2	18.12 ± 12.9^d^	36–115
U-Ca/creatinine-ratio	0.18 ± 0.11	0.24 ± 0.10	0.06 ± 0.13	0.02 ± 0.13	
U-Mg/creatinine-ratio	0.23 ± 0.13	0.27 ± 0.08	0.03 ± 0.03	0.01 ± 0.09	

Data are presented as mean ± SD or median (IQR) depending on distribution. ^a^p = 0.025, ^b^p = 0.028, ^c^p = 0.027, ^d^p = 0.003. S: serum; P: plasma; U: urine; PPI: proton pump inhibitor; PTH: parathyroid hormone.

## 3 Discussion

This study is the first to demonstrate bone affection in PPI-naïve GERD patients. We observed a significantly lower TBS compared to age- and sex-matched controls. This finding was supported by inferior BMD at all skeletal sites and higher CTX levels, although differences were not statistically significant. Accordingly, these patients may have increased fracture risk before initiation of PPIs. Treatment with pantoprazole for three months did not induce alterations in BMD, TBS or bone markers compared to controls.

We applied DXA-derived TBS, a surrogate marker of bone microarchitecture that has been shown to predict fracture risk independently of BMD (Hans et al., 2011). Interestingly, TBS was significantly lower in patients at baseline compared to controls. Hence, we speculate that GERD patients may have impaired bone quality independent of PPI treatment, which could partly explain the increased fracture risk observed in several epidemiological studies (Yu et al., 2011). Notably, there are indications that TBS may be particularily useful for prediction of fracture risk in some types of secondary osteoporosis (Hans et al., 2017). Whether this also applies in GERD patients with bone affection remains to be explored.

BMD also tended to be lower at baseline in patients with GERD, especially at the total hip, which supports the notion of bone affection in these patients not related to PPI use. There are no previous reports on BMD in PPI-naïve GERD patients, possibly because most of them have been treated with PPI before they are referred to hospital. Larger studies are warranted to assess bone parameters in patients with GERD and to explore if the increased fracture risk observed in PPI users is caused by preexisting underlying disease.

The bone affection in GERD could be attributed to several factors. The immune system is recognized as one of the most important regulators of bone turnover and is involved in bone impairment both in postmenopausal osteoporosis and secondary osteoporosis. Notably, pro-inflammatory factors, such as mucosal interleukin-6 and -8, leukocytes and oxidative stress, have been demonstrated to be involved in GERD (Yoshida, 2007). Accordingly, we found that GERD patients had significantly higher blood leukocytes and ESR at baseline compared to controls, which may reflect subclinical systemic inflammation (D'Amelio and Sassi, 2016). We observed a trend towards higher CTX in GERD patients at baseline than in controls. This could be attributed to inflammation, which has been shown to stimulate bone resorption. There is, however, limited evidence for GERD evoking a systemic inflammation. Pronounced reflux could in some patient groups cause airway inflammation, a hypothesis supported by findings from a randomized controlled trial of patients with chronic obstructive pulmonary disease, where participants treated with PPIs had fewer exacerbations (Sasaki et al., 2009). In vitro studies suggest that PPIs can exert anti-inflammatory effects that are unrelated to the inhibition of gastric acid production (Krüger et al., 2021). Unfortunately, we did not analyze ESR and leucocyte number after three months use of pantoprazole, hence a potential effect of PPI could not be evaluated.

Obesity is a major risk factor for GERD (Ness-Jensen et al., 2013), and is also associated with systemic inflammation (Tilg and Moschen, 2006). However, GERD patients and controls did not differ in BMI in the present study. GERD seems to be associated with metabolic syndrome independently of BMI (Hsu et al., 2011) and is positively associated with abdominal fat (Rattarasarn et al., 2004). Hence, waist circumference would be the preferred measure of obesity in these patients. The bone affection could also be attributed to nutritional deficiencies. Patients with GERD may avoid certain types of food that could worsen their symptoms. For example, ascorbic acid (vitamin C) has very high acidity and may induce gastrointestinal symptoms when ingested on an empty stomach. Accordingly, lower plasma levels of vitamin C have been reported in patients with Barrett's esophagitis, a condition which complicates 3 to 5% of cases of GERD (Fountoulakis et al., 2004). Vitamin C has been observed to enhance trabecular bone formation, and is essential in production of collagen in bone, thus deficiency would promote impairment of bone quality. Several studies have reported association between reduced serum vitamin C levels or low intake and osteoporosis and increased risk of fracture (Aghajanian et al., 2015).

Notably, we observed significantly lower levels of vitamin B12 in GERD patients prior to initiation of PPI, however, within the reference range. Vitamin B12 deficiency results in a rise in homocysteine, which is a risk factor for osteoporotic fractures independent of BMD (Kuroda et al., 2013). Homocysteine inhibits the action of lysyl oxidase, thereby modulating collagen cross-linking and impairing bone quality (Bailey and van Wijngaarden, 2015). PPI use has been shown to be associated with vitamin B12 deficiency (Jung et al., 2015), whereas data on vitamin B12 levels in PPI-naïve GERD patients are lacking.

Another objective of the study was to assess the skeletal effects of short-term therapy with pantoprazole, which is the most frequently used PPI in Norway. Whereas most previous studies have shown modest or no effect on BMD during PPI use (Solomon et al., 2015; Targownik et al., 2010), others found a reduction in BMD after six months, with a more marked decline in users of lansoprazole than pantoprazole (Ozdil et al., 2013). In the present study, no significant changes in BMD and TBS were observed after three months of treatment with pantoprazole. It may be claimed that treatment for three months is somewhat short to uncover effects on BMD and TBS. However, an effect of PPI on bone turnover would be expected to occur earlier than changes in BMD. Hence, the bone markers CTX and P1NP were analyzed, but no significant differences were seen after three months of pantoprazole therapy. These findings are in concordance with previous studies of patients treated with a PPI for two weeks (Kocsis et al., 2002) as well as in postmenopausal women in a randomized placebo-controlled trial evaluating effects of esomeprazole or lanzoprazole for 26 weeks (Hansen et al., 2019). A rise was seen in levels of gastrin and CgA after three months, confirming that pantopazole was taken by the patients.

The degree of acid inhibition may explain the diverse effects on BMD, esomeprazole and rabeprazole promoting a far more pronounced effect than pantoprazole (Graham et al., 2019; Graham and Tansel, 2018). In line with that, esomeprazole use was associated with a significant decline in BMD after one year of treatment, in contrast to lansoprazole, pantoprazole or omeprazole (Bahtiri et al., 2016). This concords with studies on fracture risk, rabeprazole showing the strongest association with fractures (van der Hoorn et al., 2015).

Studies of PPI users have revealed small and conflicting effects on BMD, whereas numerous studies have reported an increased fracture risk. This could be attributed to impaired bone quality not captured by BMD. Accordingly, Shin et al. showed that females exposed to PPIs displayed a lower TBS than the control group (Shin et al., 2019). Interestingly, lower TBS was associated with current PPIs use, not with recent or past PPIs usage (Shin et al., 2019).

Several mechanisms have been proposed to explain the adverse skeletal effects of PPIs. These drugs induce anacidity which has been proposed to promote malabsorption of calcium and subsequent secondary hyperparathyroidism. We found no evidence of this after three months of PPI use, which is in line with previous studies which suggest a normal calcium absorption in anacidity as long as calcium is taken with food (Hansen et al., 2010; Wright et al., 2010). Anacidity also causes hypergastrinemia and possibly also elevation of histamine levels. We and others have shown that patients with chronic atrophic gastritis, who have anacidity and a similar biochemical pattern, display lower BMD and increased fracture risk (Eastell et al., 1992; Aasarod et al., 2016c). Several rodent models support a negative effect of PPIs on bone. Omeprazole suppresses bone mineralization in rats (Cui et al., 2001). Mice deficient of the H^+^K^+^ATPase beta subunit have an osteoporotic phenotype (Fossmark et al., 2012) that is only partially rescued by a gastrin receptor antagonist (Aasarod et al., 2016b), but not by a histamine 1 receptor antagonist (Aasarod et al., 2016a). Direct effects on bone have also been proposed as omeprazole inhibits osteoblast proliferation and differentiation in vitro *(*Krüger et al., *2021**)*. In the current study, no correlation between gastrin and BMD or TBS change was observed.

The study has several limitations. It is limited by the low sample size and should be considered as a pilot study. The observation time was possibly too short to reveal effects on BMD and TBS. We examined the effects of pantoprazole only, and cannot rule out that other PPIs might affect the bone parameters differently. Moreover, no discrimination according to sex and age (e.g., post- vs. premenopausal women, > vs. < 50 years) was possible. A strength of the study is that we included PPI-naïve GERD subjects. Moreover, we studied several skeletal parameters enabling assessment of bone quantity, microarchitecture and bone turnover. Given that many patients use PPIs in a three months course, the observation time is clinically relevant. Finally, the skeletal affection in PPI-naïve GERD may suggest a possible drug prescription bias in previous studies.

In conclusion, PPI-naïve GERD patients had bone impairment that may translate to increased fracture risk. No significant changes in BMD, TBS or bone turnover markers were seen after three months of treatment with pantoprazole, indicating that short-term treatment with this PPI is safe in a skeletal health context.

## CRediT authorship contribution statement

**Kristin M. Aasarød:** Conceptualization, Investigation, Formal analysis, Writing – original draft. **Mats P. Mosti:** Formal analysis, Writing – review & editing. **Malin T. Finstad:** Investigation, Writing – review & editing. **Astrid K. Stunes:** Investigation, Formal analysis, Writing – review & editing. **Reidar Fossmark:** Conceptualization, Formal analysis, Writing – review & editing. **Unni Syversen:** Conceptualization, Project administration, Writing – review & editing.

## Declaration of competing interest

None of the authors have conflicts of interests to declare.
